# CBCT in Dental Implantology: A Key Tool for Preventing Peri-Implantitis and Enhancing Patient Outcomes

**DOI:** 10.3390/dj12070196

**Published:** 2024-06-26

**Authors:** Souheil Hussaini, Michael Glogauer, Zeeshan Sheikh, Haider Al-Waeli

**Affiliations:** 1 Oral Implantology Research Institute, Block # 18 King Salman Bin Abdulaziz Al Saud St., Dubai 39695, United Arab Emirates; souheil@oimcgroup.ae; 2 Department of Dental Oncology, University Health Network, Princess Margaret Cancer Hospital, 610 University Ave., Toronto, ON M5G 2M9, Canada; michael.glogauer@utoronto.ca; 3 Faculty of Dentistry, University of Toronto, 124 Edward St., Toronto, ON M5G 1X3, Canada; 4 Department of Applied Oral Sciences, Faculty of Dentistry, Dalhousie University, 5981 University Ave., Halifax, NS B3H 1W2, Canada; zeeshan.sheikh@dal.ca; 5 Department of Dental Clinical Sciences, Faculty of Dentistry, Dalhousie University, 5981 University Ave., Halifax, NS B3H 1W2, Canada; 6 Biomedical Engineering, Faculty of Medicine, Dalhousie University, 5981 University Ave., Halifax, NS B3H 1W2, Canada

**Keywords:** trust in physicians, CBCT, patient satisfaction, implant dentistry

## Abstract

(1) Introduction: Trust is a cornerstone of the patient–physician relationships. Unforeseen complications in the health care system could jeopardize patients’ trust in their physicians. (2) Aim: This article presents a quantitative figure regarding foreseeing the necessity of a three-dimensional quantitative visualization of bone structure and concurrently preparing for an ancillary procedure by a dentist to successfully perform the surgery that could minimize unforeseen complications; (3) Materials and method: This retrospective study has been derived based on an analysis of 1134 patients who had received 4800 dental implants from January 2001 to August 2020, out of which 200 cases were randomly selected for this study. Each procedure during implant treatment was categorized as OPG (Orthopantomography) or OPG with CBCT as per all the procedures which included and were coded as follows, 1: Surgery & Restoration, 2: GBR (Guided Bone Regeneration), 3: GTR (Guided Tissue Regeneration), 4: Block Bone Graft, 5: Spreading, 6: Splitting, 7: Internal Sinus, 8: External Sinus, 9: PRF (Platelet Rich Fibrin). Any of the 200 cases in which implant placement could not have been performed for reasons related to a lack of CBCT were selected for this study. The surgery was aborted halfway through without implant placement in these cases due to a lack of bone quantity and/or lack of primary stability. These cases were registered for re-evaluation and statistical analysis; (4) Results: 7% of the cases that used OPG alone led the surgeon to unexpectedly abort in the middle of the surgery without implant placement. All (100%) of the patients who had CBCT during treatment planning were able to receive implants during the surgery. None of the patients left the surgery without receiving implants if CBCT was used (0%); (5) Discussion: Radiographic image quality is defined as the amount of information within the image that allows the radiologist to make a diagnostic decision with a particular level of certainty (Martin et al., 1999) and hence the importance of CBCT. The unexpected 7% of devastating situations for patients who started surgery but did not have implant placement led to [A] aborting the surgery, [B] procedural difficulties requiring an alternative treatment plan, [C] a negative impact on the patient’s behavior, and [D] wanting to change doctor due to a lack of trust; (6) Conclusion: This study indicates that in implant dentistry patients’ mistrust could be avoided by 7% if CBCT is obtained. It also shows the significance of cone-beam computed tomography as an adjunct to panoramic radiography during the diagnosis and treatment planning phase. The use of panoramic radiography alone can lead to a 7% likelihood of misdiagnosis. A lack of CBCT during treatment planning negatively affects the outcome of surgical procedures.

## 1. Introduction

Patient-physician trust is crucial, yet its psychological impacts, such as losing trust in a physician, are less quantified than physical consequences. One significant but often unspoken aspect of patient-physician trust is the ability to save the patient time and money. If properly diagnosed and documented, additional procedures should not be avoided solely to increase the Patient Trust Scale, as this could inadvertently lead to a higher complication rate. 

Even though radiography has evolved from a traditional intraoral periapical (IOPA) technique to more sophisticated methods like orthopantomography, computerized tomography and cone-beam tomography [1], the importance of a CBCT is still in a controversial stage.

The lack of a third dimension and orthogonal sectional images was met with the integration of CT (computed tomography) in the early 1990s [2]. Some of the disadvantages of CT include equipment cost and the space required for installation, which was alleviated by the development of cone-beam CT (CBCT). CBCT also solved the main disadvantages of increased radiation dose as seen in the advanced computerized tomographic method [3].

Conventional methods like IOPA and OPG are still considered satisfactory protocols for providing successful treatments in many clinical situations. However, the larger question is whether computerized tomography or CBCT are mandatory for all implant placements and evaluations, especially keeping in mind the large radiation dose and cost. Directives and position statements have been placed by various bodies and associations like the American Academy of Oral and Maxillofacial Radiology [4], so detailed risk and benefit analysis is essential while advising on these procedures. 

The anatomical variations in different parts of the oral cavity dictate various considerations in dental implant treatment planning [5]. These variations affect the diagnosis, surgical procedure, impression technique, restoration modality, radiographic requirement, and maintenance approach to long-lasting treatment. However, most published literature classified that the jawbone was related to ordinal bone quality and quantity based on panoramic and cephalometric radiographs. In relation to bone quality: In 1985, Lekholm & Zarb described a classification method to pre-operatively score bone density based on panoramic radiographs [6]. This classification was used worldwide because it is easy to use and does not involve a huge investment. Bass & Triplett showed in their results on 1097 Brånemark implants in 303 jaws that bone density grade four (4) exhibited the greatest failure rate [7]. Jaffin & Berman also found a high failure rate (35%) in type 4 bone [8]. All these studies investigated bone density in relation to outcome when implants were placed according to standard protocol [9]. The results of these studies show that the early loading of implants utilizing a flapless surgical technique with CT-guided surgical stents may be possible [10]. In 1985, Misch and Judy established four basic divisions of available bone for implant dentistry in the maxilla and mandible, which also follows the natural resorption phenomena [11]. The implant treatment options for each division were also presented. These four divisions were then expanded to six categories in order to extend the specific organized approach to implant treatment options for surgery and prosthodontics. 

The introduction of CBCT imaging to surgical implant treatment planning has allowed clinicians to make measurements in dimensions not previously available. Today, the accuracy in placing implants or surgical osteotomies in close proximity to vital structures is more frequently dependent on the diagnostic measures taken from these imaging modalities [12].

There is a large body of literature on the accuracy of measurements made on CBCT images, the results of which are variable. Several investigators have reported that linear measures made on CBCT are sufficiently accurate for use in pre-surgical planning [13]. 

Another distinct advantage of CBCT technology is the ability to plan implant therapy virtually, with the use of specifically designed 3D software [14]. In the last decade, several computer-based software programs for pre-surgical implant planning have been developed that utilize images derived from CBCT. Using these programs, clinicians could select and virtually “try in” implants of different diameters and lengths in order to select the implant best suited for a given location [15,16]. In 2000, rapid prototype medical modelling manufactured from CBCT data became available to the dental profession. With this advancement, information from CBCT images could be transferred directly to the patient via a prefabricated surgical stent [17]. It has been suggested that CBCT guided surgery is superior to non-CT guided surgery due to its potential to eliminate errors with manual placement [18]. The use of software systems with CBCT imaging has become one of the primary tools for dental pre-surgical implant treatment planning [14]. Not only can one select for a particular implant size and length, but alveolar ridge height and width as well as the proximity of adjacent anatomic structures can be determined. Areas of inadequate ridge height or width can be identified and then considered for ridge augmentation procedures [12]. When used in combination, traditional 2D images and CBCT provide increased information and improved detail [1].

Despite the integration of CBCT in clinical practice, bias still exists towards conventional films due to the fact that most clinicians are more acquainted with the use of these imaging methods [14]. Even then, there are clinical situations that are ideal for one to proceed with implants, availing information obtained from OPG or other two-dimensional methods alone.

The choices that have to be finalized prior to surgery based mainly on radiographic findings include the type of implant, number of implants, anatomical consideration, cost, time required, number of procedures, and other required investigations [17]. If the obtained radiograph runs too short to visualize the information required for the entire procedure, it would be devastating after minutes of surgery if the surgery could not be completed and was jeopardized. The effects of such an event could be irreversible physically and psychologically for both the patient and the dentist performing the surgery. Furthermore, the radiographs taken to arrive at a choice that would be reflected in the final outcome and basic objective of providing successful and sustainable implant prosthesis are of vital importance [13,14].

This study addresses the necessity of using OPG vs. cone-beam CT by quantitative means.

The purpose of this research is to clarify for the person performing an implant surgery how often the planned cases cannot be performed without CBCT investigation to complete the surgical procedure.

## 2. Aims of the Study

The aim of the study is to determine the efficacy of conventional cone-beam computed tomography [CBCT] as an adjunct to panoramic radiography in the prediction of successfully following a treatment plan. 

### 2.1. Hypotheses

#### Hypothesis

**H0:**  *There is no significant difference between using CBCT as an adjunct to OPG in the prediction of successfully following a treatment plan.*

**H1:**  *There is a significant difference in using CBCT as an adjunct to OPG in the prediction of successfully following a treatment plan.*

## 3. Materials and Methods

### 3.1. Research Design 

This is a retrospective and analytical study of the clinical records of 1134 patients who were successfully treated with dental implants and had been using them for more than 6 years post operatively with satisfactory structural and functional concerns. From a database of 1134 patients who received 4800 dental implants between January 2001 and August 2020, a sample of 200 files were randomly selected using Research Randomizer—an online resource that generates random numbers to experimental conditions—and then divided into two groups accordingly. Hence, we had no knowledge of what type of cases had been selected. A prosthodontist with no knowledge of the goal of the study was asked to randomly select 200 files from a medical facility database in two separate sessions from two separate branches of one dental group (100 each) and present them for this study. These first 100 files (sample 1/Branch A) had received panoramic and cone-beam computed tomography (CBCT) (Appendix A) and the second 100 (sample 2/Branch B) were collected from patients who had received only panoramic radiographs during their diagnostic visit in a clinic that did not have CBCT inhouse, did not provide CBCT by default, and sent patients for CBCT imaging on a case-by-case basis. There were no exclusion criteria for the part of the mouth or the dentist’s level of competency because all clinicians used the same protocol for implant placement. The sample was analyzed by the Students’ *t*-test and Mann–Whitney test (Appendix B). 

Number of procedures: Each procedure during implant treatment received a code as follows: 1: Implant Placement, Surgery & Restoration of the Implant, 2: GBR, 3: GTR, 4: Block Bone graft, 5: Spreading, 6: Splitting, 7: Internal Sinus, 8: External Sinus, 9: PRF (Illustrations 1 and 2). Each of the 200 cases was examined in detail. The total number of procedures for each case was calculated and then divided by the number of cases in each category to determine the average number of procedures per group. 

### 3.2. Statistical Analysis 

**Figure 1:**
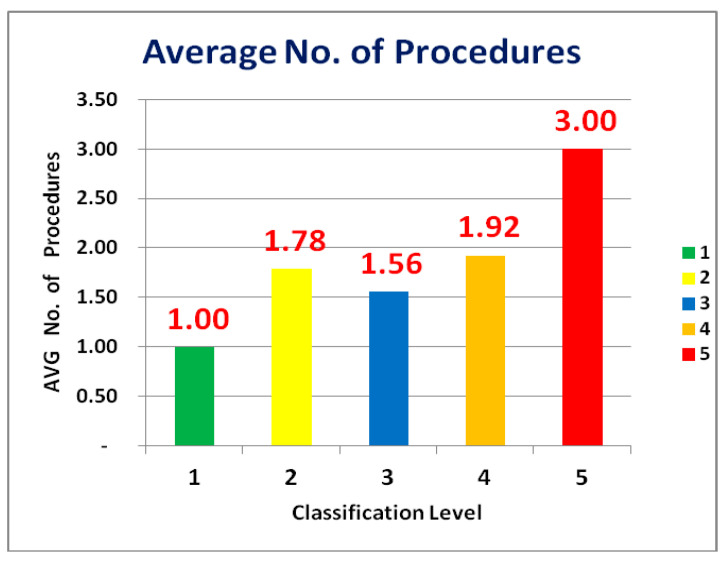
This graph visually represents the data from Table 1 being the average number of procedures according to the location [groups 1–5] from Sample 1 and 2 in different areas in the mouth.

The results of implant placement with and without CBCT (Table 1 and Figure 1) after CBCT protocol (test group, sample 1) showed lower values when compared to only panoramic cases for failure in implant placement (control group, sample 2) (*p* < 0.05; One-Way ANOVA test). Therefore, when comparing the control with the test group, CBCT vs. only panoramic showed a significant difference (*p* = 0.00714).

An inferential and qualitative statistical analysis was performed by computing and using Sample Means and Sample Standard Deviation. The Students’ *t*-test was used to compare means and proportions, with inference given on the basis of the value of *p* (statistical significance was denoted when *p* < 0.05). 

The Mann–Whitney test was used to compare differences between independent classifications under minimum, maximum, and average of HFU and time. 

One-Way ANOVA was used to find significant difference between CBCT and PAN.

## 4. Results

Table 1 and Table 2 demonstrate the percentage that led to aborting the surgical procedure in 7% of the cases when using OPG alone and 0% when using CBCT. In order of the number of cases according to their location, the most implants were in the posterior mandible (64 out of 200 implants) followed by the anterior maxilla (59), posterior maxilla without requiring sinus lift (48), anterior mandible (28), and sinus lift (11) accordingly.

### 4.1. Statistical Observations

Test Statistic−2.6933 (ratio between the difference to the standard error)*p*-Value<0.05 (*p* = 0.00714) 

### 4.2. Statistical Inference

There was a significant difference between predictability in CBCT and predictability in PAN.

The Z-Score was −2.6933. The *p*-value was 0.00714. The result was significant at *p* < 0.05. 

The Z Statistic was used for the Large Sample Test and the T Statistic for the Small Sample Test. 

The proportion for Observation 1 was 0. The proportion for Observation 2 was 0.07.

## 5. Discussion

As compared to 2D radiographic techniques, CBCT [Appendix C] imaging provides additional information [1,2,9,14] but with the disadvantage of an increased radiation dose to the patient [3]. The major issue in this study was the confrontation of unexpected ridge topography [19,20,21,22,23,24,25,26] which led to aborting the surgery in 7% of the cases performed using OPG alone and 0% when using CBCT. This unforeseen situation led to a lot of procedural difficulties, including the need for an altered treatment plan which may have had a negative impact on the patient [27]. In the selection of a pre-surgical imaging modality, clinicians must consider the ability of the radiographic technique to have adequate diagnostic quality to clearly depict the landmarks of interest. Selection of a diagnostic technique with higher patient exposure may be necessary to achieve the desired surgical results [28]. 

**Figure 2:**
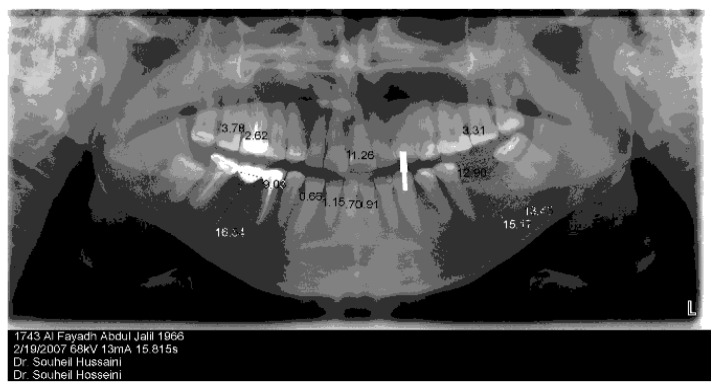
A panoramic radiograph demonstrating a radio-opaque marker.

Due to the image distortion produced in panoramic images [29,30], they must be used cautiously when measuring bone, especially when attempting to determine bone height [31,32,33]. Tronje et al. (1981) suggested that when greater accuracy is required, measurements on orthopantomograms are not recommended [34]. The fact that panoramic images have inherent magnification is generally appreciated. To determine the exact magnification in a particular area, reference objects with known dimensions are placed in situ when taking an image [35,36]. A true magnification can then be calculated from the ratio of the projected to true length of the reference object [37]. This resultant magnification was taken into consideration when orthopantomograms were used for surgical planning in all the cases in this study by the use of a 14 mm long and 2.5 mm wide radio-opaque marker with these images from which an accurate determination of all measurements was obtained (Figure 2). 

One person was designated to analyze the CBCT and OPG radiographs. The results of the previous studies by Berco et al. 2009 [38] demonstrated that those observers with less experience reading orthopantomograms and CBCT images did not perform any differently than the more experienced clinicians with paired *t*-test comparisons demonstrated no statistically significance difference between groups. Contrary to this, one study has reported that the major influence on reliability of a landmark is inter-observer variation and that this can affect overall accuracy outcomes [39]. Most purchasers of CBCT scanners are specialist dentists and maxillofacial surgeons and not radiologists [40]. Currently, in regions where CBCT technology is in regular use, images are read by clinicians at various levels of training and not necessarily by radiologists [34,41,42,43,44,45]. One study reported that a standardized period of training reduced structure-related causes of variability among observers [46]. In our study, one prosthodontist specialist with experience performed all readings of the entire study randomly, without knowing the age, gender, or status of the dentition; when the two groups were selected, we did eliminate bias in their selection, so there was no bias toward any area of the mouth nor to the images that might improve accuracy while being measured. In regions where cone-beam computed tomography (CBCT) technology is routinely utilized, it is common for images to be interpreted by clinicians with varying levels of training rather than exclusively by radiologists. This practice highlights the critical need for comprehensive training and standardization in the interpretation of CBCT images. Inadequate training can lead to misinterpretations, potentially compromising diagnostic accuracy and treatment outcomes. Therefore, it is imperative to establish rigorous training programs and standardized protocols for all clinicians involved in CBCT interpretation. By doing so, we can ensure that clinicians possess the necessary skills and knowledge to accurately interpret CBCT images, thereby enhancing diagnostic precision and improving patient care. The article addresses this necessity, emphasizing the role of structured training in optimizing the utilization of CBCT technology in clinical practice.

Prediction of an ancillary procedure in pre-surgical planning was more knowable with the use of CBCT [17]. In the present study, the ability to predict the ancillary procedures vis-à-vis the quantity of bone graft required, angle of splitting the bone, buccopalatal width of the maxillary sinus, etc. assisted in the armamentarium required for each surgery [5,47,48,49,50,51]. Resorption of bone, position of the IAN canal, and the location of the maxillary antrum have been reported as being more clearly depicted on CBCT than on conventional films [52,53,54]. Provisional guidelines for the use of CBCT were created by the SEDENTEXCT project in 2009. These evidence-based guidelines include referral criteria, quality assurance guidelines, and optimization strategies [55]. The American Academy of Oral and Maxillofacial Radiology defends the position that the success of any surgical treatment is, in part, dependent on adequate diagnostic information about the bony structures of the oral region, including accurate linear measurements [13,56]. There is no question that a combination of traditional films and CBCT together provide the best anatomic detail [57], but orthopantomograms alone can be invaluable in identifying the need for further imaging studies [58].

The total number of ancillary procedures during implant placement was slightly higher (6%) when CBCT was taken. This could be due to the fact that during radiographic observation a better visualization could perhaps have caused more planning with more detailed ancillary procedures for the betterment of the case. Suomalainen et al. (2008) [59], upon linear measures made on CBCT images of both height and width, found them to be reliable and accurate when compared with those of the cadaveric mandibles. They concluded that CBCT should be considered as a reliable tool for implant planning [14]. 

Cone-beam computed tomography with pre-surgical planning caused more ancillary procedures to be performed during implant placement surgery [13,14]. Early implant placement with simultaneous contour augmentation using CBCT in a study performed by Dannie Buser, et al. (2013) [60] demonstrated stable peri-implant hard and soft tissues for all 41 implants examined and satisfactory aesthetic outcomes overall [10,61]. Follow-up at 5 to 9 years confirmed again that the risk of mucosal recession was low with early implant placement [62]. In addition, contour augmentation with guided bone regeneration was able to establish and maintain a facial bone wall in 95% of patients [63]. Studies have demonstrated the efficacy of endosseous implants in various clinical scenarios highlighting the long-term success rates and potential complications associated with these procedures offering valuable insights into their longevity and performance over time in clinical practice and similarly in continuing education, emphasizing the importance of the precautionary context clinical practice model [64,65,66]. Similarly, in our study we found that more ancillary procedures were performed when CBCT was available vs. fewer ancillary procedures being performed with OPG alone—61% with CBCT/56% without (a 6% difference). Radiographic image quality is defined as the amount of information within the image that allows the radiologist to make a diagnostic decision with a particular level of certainty [67].

### Clinical Implications

The adjunct use of CBCT is merely a means of allowing practitioners to decide if proposed treatments are within their abilities, and to ensure that patients are in agreement with the proposed outcomes of treatment [14]. This leads to: (1) better patient acceptance, (2) improved professional communication, (3) enhanced insurance reimbursement, (4) standardized criteria for outcome assessment, (5) preparation for materials and armamentarium requirement and finally as an instructor or clinic director, and (6) a better screening tool to assist dental schools with patient admission. 

It is good to inform the patient at an early stage of treatment planning that the necessity of acquiring CBCT imaging is related to location, and the need (or not) for bone augmentation [14]. The patient can make that decision knowingly, so the doctor can to a certain extent insist on the CBCT requirement accordingly as the level of complexity increases, for legal and diagnostic purposes. 

Recognizing patient anger is important for keeping health care providers safe [68]. If the dentist is under severe stress because the procedure was not successful, this could affect the patient’s feelings, and could translate in the patient’s mind to negligence, resulting in anger and aggression [69] Although uncommon, extreme instances of violence directed at health care providers do occur. In 2010, Warren Davis shot his mother’s orthopedic surgeon before killing his mother and himself after he learned that back surgery had left his mother paralyzed [70]. In 2014, a strikingly similar situation occurred at Sacred Heart Hospital in Cebu City, Philippines, when a wheelchair-bound patient, who was upset about not being able to walk after spinal surgery, shot and killed his orthopedic surgeon before killing himself [71]. In 2015, Stephen Pasceri shot and killed Dr. Michael Davidson, a Brigham and Women’s Hospital cardiologist, whom Mr. Pasceri believed had inappropriately prescribed his mother an antiarrhythmic agent, which he was convinced had contributed to her death [72]. While extreme acts of violence are uncommon, violent crimes in health care institutions have increased significantly in recent years [73].

Recognizing patient anger is also necessary for ensuring patient safety. Partially in response to the increase in violent incidents, in 2014, 52% of medical centers in a national survey reported that they allowed security guards to carry guns [74]. 

Beyond physical harm, there are other consequences of the failure to identify and appropriately respond to angry patients. Anger disrupts the doctor–patient relationship and can cause patients to miss their next appointments, to be less adherent with their medications and home care instructions, and to develop overall worse health outcomes [75,76].

Additionally, anger contributes to many malpractice lawsuits. When physicians and hospitals address patient anger by admitting mistakes and apologizing, the rate of malpractice lawsuits declines [77]. Since lawsuits decline when physicians apologize, it is reasonable to surmise that such suits are often driven more by anger than the actual outcome.

In Oral medicine and Radiology it helps in diagnosing and framing a treatment plan of difficult cases by easy access to various specialists through transfer of radiologic images of lesions. In maxillofacial surgery tele-dentistry may be helpful for appropriate treatment of complicated cases by analysis of advanced dental imaging techniques (like CBCT) which are often not available in one center [78,79]. 

The first CBCT procedure codes were published in 2009 (a code does not in itself assure insurance coverage because it depends upon the contract between the insurance company and the patient however, the absence of a code makes claims for payment problematic). The inclusion of addition codes heralds the acceptance and description of various aspects of CBCT imaging as well as the emergence of additional imaging modalities as valid and reliable technologies with bona fide evidence based advantages in dentistry. Courses of study in post-graduate OMFR training should not only reflect familiarity with these techniques but competent expert knowledge of the basic sciences as well as clinical proficiency associated with these advanced imaging modalities [80].

Cone-beam computed tomography has provided useful tools for assessing outcomes of oral rehabilitation with dental implant, whereas some dentists are unaware of their clinical benefits resulting from their non-use [81].

Barriers to including implant placement surgery in the undergraduate curriculum include limited time, lack of trained faculty staff, investment costs and patient recruitment [29,82,83]. Nevertheless, educational programs that provide some kind of (pre-)clinical experience have reported positive outcomes. Pre-patient care laboratory exercises were reported to have a positive influence on future plans to perform implant therapy [84]. Participating in an elective course on treating selected edentulous and partial edentulous patients, including the participation in diagnosis, treatment planning, surgical placement and prosthodontics procedures, appeared to be strongly correlated with offering and restoring implants after graduation [85]. When comparing students from a dental school with and without extensive implant education (laboratory and clinical experience to place and restore implants), graduates with clinical experience had twice as many implant restorations in their practice, placed more dental implants, referred more patients for specialized surgery and more often followed continuing education in implant dentistry [86]. When students were asked additional questions on the actual implant surgery pointed out that reading of CT scans was easy to cope with [87].

With substantial evidence indicating that maintaining a minimum of 2 mm of buccal bone significantly reduces the incidence of peri-implantitis, this article indirectly establishes the relationship between the use of CBCT and the prevention of peri-implantitis through a linear correlation. By providing precise three-dimensional visualization of the bone structure, CBCT aids in achieving optimal treatment planning and implant placement, thereby reducing the risk of peri-implantitis [88].

## 6. Limitations of the Study with Justification

The correlation between the number of ancillary procedures performed when a CBCT was in hand vs. the cases that was done with only a panoramic radiography, the evaluation of quantitative disappointment of the patients after not receiving their implant as promised, the probability of these cases could have been higher or lower if the entire 4800 implants were evaluated. With diseases of low incidence, the controlled retrospective study may be the only feasible approach. Comparing an observational study vs. a controlled retrospective study, the latter category was used to include collection of data to elaborate differences in incidence and mortality which appear when edentulism can be defined as a disease and classified newly available information. 

## 7. Conclusions

This study indicates the significance of cone-beam computed tomography [CBCT] as an adjunct to panoramic radiography in the prediction of success following the treatment plan and outcomes. Beyond physical harm, there are other consequences of the failure to identify and appropriately respond to angry patients. Anger disrupts the doctor–patient relationship and can cause patients to miss their next appointments, to be less adherent with their medications and home care instructions, and to develop overall worse health outcomes.

The percentage that led to aborting the surgery was 7% of the cases undertaken using OPG and 0% when CBCT was used. Considering that, further preclinical and clinical studies are necessary to determine how different diagnostic tools and protocols can affect implant placement with different treatment modalities.

## Figures and Tables

**Table 1:** Average number of procedures according to the location [groups 1–5] (Sample 1 and 2) required to complete the surgical procedure per classification in different areas in the mouth.

Classification (Group 1–5)	Sum of No. of Procedures (Sample 1)	No. of Cases (Sample 1)	Average No. of Procedures per Group (Sample 1)	Sum of No. of Procedures (Sample 2)	No. of Cases (Sample 2)	Average No. of Procedures per Group (Sample 2)	Average No. of Procedures per Group (Sample 1&2)
1	14	14	1.00	4	4	1.00	1.00
2	37	19	1.95	47	29	1.62	1.78
3	53	33	1.61	47	31	1.52	1.56
4	65	31	2.10	49	28	1.75	1.92
5	9	3	3.00	24	8	3.00	3.00
Total	178	100		171	100		

**Table 2:** Average Number of Implants Placed in Different Areas of the Mouth [groups 1–5].

ONE WAY—ANOVA						
Groups	Count	Sum	Average	Variance		
1 Anterior Mandible	14	9165	654.64	11,006.52		
2 Posterior maxilla without Sinus lift	19	10,046.5	528.76	119,218.37		
3 Posterior Mandible	33	19,778	599.33	21,139.45		
4 Anterior Maxilla	31	17,415	561.77	67,595.95		
5 Posterior Maxilla with Sinus lift	3	639.5	213.17	18,907.58		
significant at *p*-value < 0.05						
**ANOVA Table**						
Source of Variation	SS	df	MS	F	*p*-value	F crit
Between Groups	545,073.82	4	136,268.46	2.57	0.0426	2.47
Within Groups	5,031,171.32	95	52,959.70			
Total	5,576,245.14	99				

## Data Availability

The data presented in this study are available on request from the corresponding author due to privacy.

## Appendix A

**Table A1:** Technical data.

Anode Voltage	60–90 kV
Anode current	1–14 mA
Focal spot	0.5 mm, fixed anode
Image detector	Flat panel
Image acquisition	Single 200 degree rotation
Scan time	7.5–27 s
Reconstruction time	2–25 s

Planmeca ProMax 3D was used for CBCT and OPG

Planmeca Oy, Asentajankatu 6
FIN-00880 Helsinki, Finland
Tel. +358 20 7795 500
Fax. +358 20 7795 555
e-mail: firstname.lastname@planmeca.com

## Appendix B

Protocol for implant placement

1. Pre-operative CBCT prioritizing Screw Retained Prosthesis.
2. Implant size selection performed using the treatment planning checklist.
3. Equal Distance mesially and distally at three reference points; [coronal, middle, apical] while the distance should not be <1.5–2 mm space to the tooth and 3 mm space between Implants.
4. All implants should be parallel with each other while being placed 4 mm apical to gingival line preferably with 1 mm sub-crestal.
5. 45 Ncm (+5/−10) Torque at the time of Implant placement.
6. Extraction to be performed with periotomes keeping surrounding teeth, prosthesis, and bone intact. Documented 2 mm buccal bone, with or without bone augmentation.
7. Time spent in ant. mandible 19, Post Max 29, Post mandible 36, ant. maxilla 42 and implant sinus lift 50 average with + 10 min.
8. Implants placed in line with functional cusp areas and corresponding occlusal contacts.
9. Good documentation of case with all details and photography documented using the Logbook.
10. Explaining to the patient the importance of annual maintenance and probability of peri-implantitis in written informed consent using the restoration checklist.

[Appendix A was added to clarify the protocol-based unified approach in all cases]

## Appendix C

Bone density based on HU values given by Misch:

D1: >1250 HU
D2: 850–1250 HU
D3: 350–850 HU
D4: 150–350 HU
D5: <150 HU
